# The whole genome dataset of *Ichthyscopus pollicaris*

**DOI:** 10.1016/j.dib.2024.110704

**Published:** 2024-07-04

**Authors:** Tianxiang Gao, Wenyu Li, Yinquan Qu, Xingle Guo, Yiting Wang, Chenfeng Zhao, Fangrui Lou, Qi Liu

**Affiliations:** a Fishery College, Zhejiang Ocean University, Zhoushan, Zhejiang 316022, China; b School of Ocean, Yantai University, Yantai, Shandong 264005, China; c Wuhan Onemore-tech Co., Ltd, Wuhan, Hubei 430000, China

**Keywords:** *Ichthyscopus pollicaris*, Genome, Third generation sequencing, Phylogenetic relationship

## Abstract

The classification of the Uranoscopidae species is controversial and the *Ichthyscopus pollicaris* belonging to Uranoscopidae was first reported in 2019. In the present study*,* the whole genome sequence of *I. pollicaris* were generated by PacBio and Illumina platforms for the first time. After *de novo* assembly and correction of the high-quality PacBio data, a 527.25 Mb I. pollicaris genome with an N50 length of 11.25 Mb was finally generated. Meanwhile, 170.41 Mb repeating sequence, 21,263 genes, 784 miRNAs, 2,225 tRNAs, 3004 rRNAs, and 1422 snRNAs were annotated in *I. pollicaris* genome. Furthermore, 3,168 single-copy orthologous genes were applied to reconstructed the phylogenetic relationship between *I. pollicaris* and other 11 species. The draft genome sequences have been deposited in NCBI database with the accession number of PRJNA1071810.

Specifications Table

**Table:** 

Subject	Biological Sciences / Omics / Genomics
Specific subject area	Phylogenetics and bioinformatics of *Uranoscopidae species*
Data format	Raw and analysed
Type of data	Table, Image, and Figure
Data collection	The PacBio and Illumina HiSeq2500 platforms were used to sequence the whole-genome data of *I. pollicaris*. RepeatMasker software, RepeatProteinMask software, LTR_Finder software and *de novo* prediction method were applied to predicted the repeating sequences. The bwa, minimap2, BUSCO, samtools, picard and GATK software were applied to evaluate the assembly effect of the genome. The non-coding RNAs (including miRNA, tRNA, rRNA, and snRNA) were annotated by the tRNAscan-SE, Infernal, and BLASTN softwares. OrthoMCL software was applied to obtained the single-copy orthologous genes. Finally, the phylogenetic tree was constructed with RAxML software.
Data source location	Institution: Zhejiang Ocean University, Wuhan Onemore-tech Co., Ltd City: Zhoushan, Wuhan Country: China
Data accessibility	Raw sequences of Ichthyscopus pollicaris Repository name: SRA NCBI Data identification number: PRJNA1071810 Direct URL to data: https://www.ncbi.nlm.nih.gov/bioproject/PRJNA1071810

## 1 Value of the Data

• The genome provided in the present study is necessary for species identification and phylogenetic relationship study of Ichthyscopus pollicaris.
• The genome sequences can improve the genetic information of Uranoscopidae species and provided reference information for the whole-genome assembly of other Uranoscopidae species.
• The whole-genome sequences can provide reference information for future studies of population genetics and habitat adaptive evolution of I. pollicaris.

## 2 Background

The phylogeny of Uranoscopidae species is more complex. There are considerable differences between the phylogenetic results based on morphological and molecular features. I. pollicaris was previously confused as I. lebeck, and was accurately described in 2019 [1]. The present study obtained the whole genome information of *I. pollicaris*, and then more precisely constructed the phylogenetic relationship of Uranoscopidae based on single-copy orthologs.

## 3 Data Description

**Fig. 1:**
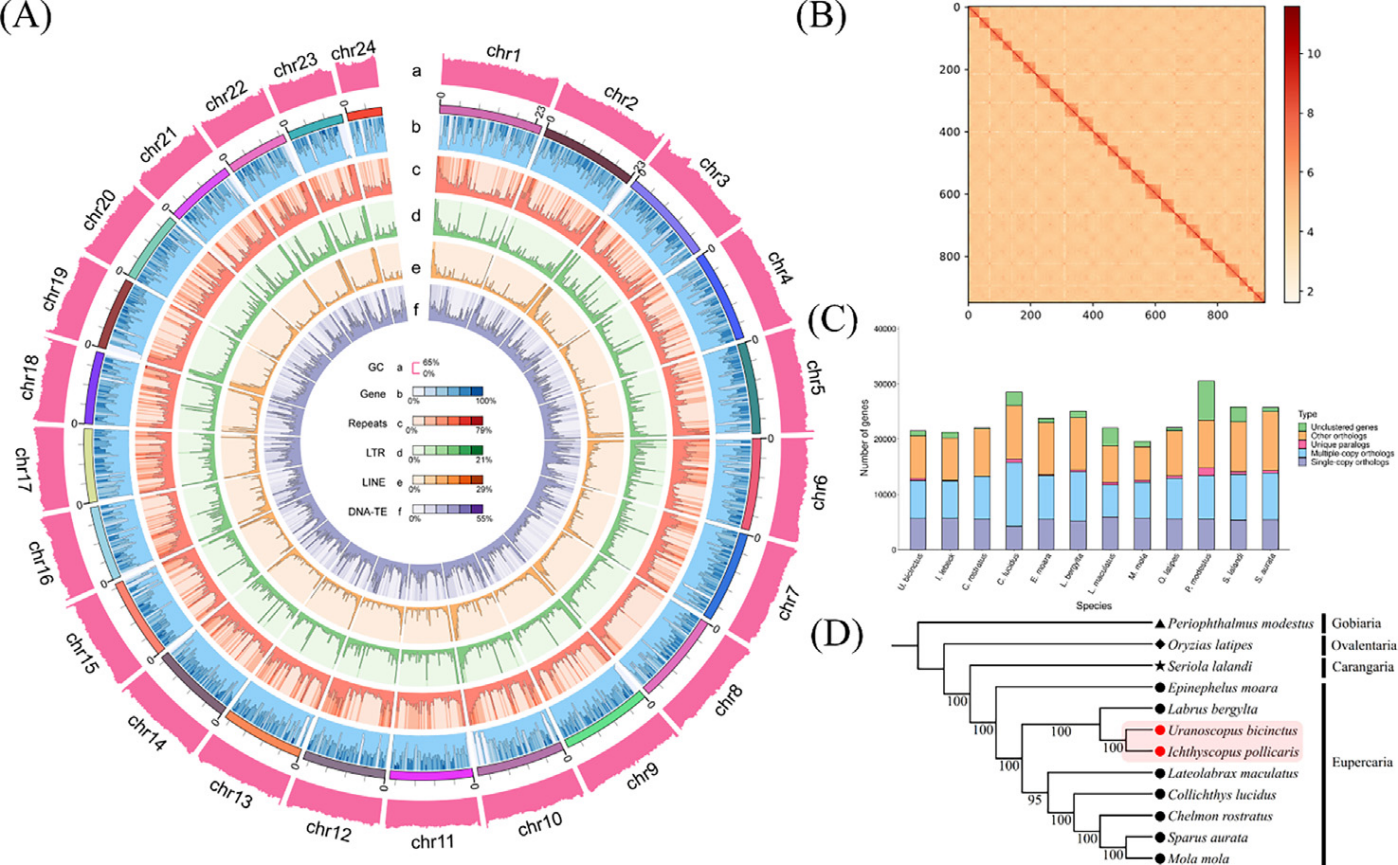
(A) Genome circle diagram of *I. pollicaris*. (B) Clustering heat map of Hi-C. (C) Statistical results of homologous gene number of selected species. (D) The phylogenetic tree reconstructed using single-copy orthologous genes of the *I. pollicaris* and other 11 selected fish species.

In the present study, the PacBio and Illumina platforms were used to sequence the whole-genome information of I. pollicaris (Zhoushan, China). A total of 49.07 Gb of high-quality PacBio reads (https://www.ncbi.nlm.nih.gov/sra/SRX23734126) were applied to *de novo* assembled, and a 562.68 Mb I. pollicaris genome was obtained. The above genome was corrected, deredundancy, and chromosome constructed using 84.43 Gb Hi-C data, a 527.25 Mb I. pollicaris genome was eventually generated, with scaffold N50 length of 20.42 Mb and contig N50 length of 11.25 Mb (Table 1). Meanwhile, 97.08 % complete BUSCOs were covered by genome sequences. The comparison rate of PacBio reads, Illumina reads, repetitive sequence content, GC content, heterozygosity, proportion of homozygous SNP (Single nucleotide polymorphism), homozygous InDel (Insertion and deletion), heterozygous SNP, and heterozygous InDel on the I. pollicaris genome were 99.13 %, 98.73 %, 23.18 %, 43.11 %, 0.41 %, 0.002 %, 0.006 %, 0.193 %, and 0.104 %, respectively (Fig.1. A). Combining RepeatMasker software [2], RepeatProteinMask software [2], LTR_Finder software [3] and *de novo* prediction method, a total of 170,413,431 bp repeating sequence was ultimately predicted. Furthermore, 21,263 genes were predicted, of which 19,639 were obtained functional annotation information (Table 2). Additionally, 784 MiRNAs, 2225 tRNAs, 3004 rRNAs and 1422 snRNAs were also predicted in the currently published I. pollicaris genome (Table 3). In conclusion, we characterized a high-quality reference genome of I. pollicaris and these sequences can provide a useful resource for exploring the biological processes of I. pollicaris.

**Table 1:** Summary of the assembled genome of *I. pollicaris*.

Mode	Total length	Total number	Max length	N50	N90
Assembly	562,679,698	744	25,126,692	10,964,685	254,152
Assembly+ racon	562,879,681	715	25,167,036	10,981,672	255,824
Assembly+ racon+pilon	562,651,429	715	25,153,247	10,976,673	256,056
Assembly+ racon+pilon+redundans	527,782,741	413	25,153,247	11,294,608	621,734
Assembly+ racon+pilon+redundans+Hi-C	527,249,938	503	25,436,915	20,420,678	8630,076

**Table 2:** Statistical results of function gene annotation of *I. pollicaris*.

	Database	Number	Percent (%)
Total		21,263	
Annotated	InterPro	19,101	89.83
	GO	14,579	68.57
	KEGG ALL	9095	42.77
	KEGG with KO	6727	31.64
	Swissprot	5648	26.56
	TrEMBL	10,864	51.09
	TF	745	3.50
	Pfam	18,434	86.70
	NR	11,120	52.30
	KOG	17,258	81.16
	Total	19,639	92.36
Unanotated		1624	7.64

**Table 3:** Statistics of non-coding RNA annotation results of *I. pollicaris*.

Type	Copy	Average length(bp)	Total length(bp)	% of genome
MiRNA	784	86	67,608	0.012823
tRNA	2225	74	165,115	0.031316
rRNA	3004	208	625,158	0.118570
18S	176	1665	293,074	0.055585
28S	0	0	0	0.000000
5.8S	135	153	20,685	0.003923
5S	2693	116	311,399	0.059061
snRNA	1422	153	217,422	0.041237
CD-box	184	141	25,887	0.004910
HACA-box	76	150	11,392	0.002161
splicing	1155	155	178,449	0.033845
scaRNA	7	242	1684	0.000321

The whole-genome sequence of I. pollicaris was further applied to the phylogenetic analysis of I. pollicaris and other 11 species (including Periophthalmus modestus, Seriola lalandi, Oryzias latipes, Uranoscopus bicinctus, Collichthys lucidus, Labrus bergylta, Epinephelus moara, Lateolabrax maculatus, Sparus aurata, Mola mola, Chelmon rostratus). The phylogenetic tree based on 3168 single-copy orthologous genes showed that the I. pollicaris and the U. bicinctus, both belonging to the Uranoscopidae were first clustered into one branch, and then clustered together with the other five Eupercaria species. Meanwhile, the P. modestus belonging to Gobiaria was located at the root of the present phylogenetic tree (Fig. 1. C and D). Considering that the divergence of conserved single-copy orthologous genes always leads to species divergence, we strongly believe that the phylogenetic relationships of I. pollicaris based on single-copy orthologous genes can be more reliable.

## 4 Experimental Design, Materials and Methods

The *I. pollicaris* sample was collected from the coast of Zhoushan, China. Then, the *I. pollicaris* was anesthetized with MS-222, and then quickly dissected by sterile scissors and tweezers, and muscle, heart, stomach, liver, intestine, spleen, kidney, eye, brain, skin, ovaries, and blood were obtained. All tissues were separately snap-frozen in liquid nitrogen and then stored at −80 ℃. It is worth noting that the muscle was used for DNA library construction, and heart, stomach, liver, intestine, spleen, kidney, eye, brain, skin, ovary, and blood were used for RNA library construction.

High-quality genomic DNA was extracted from the muscle tissues of *I. pollicaris* using the Blood & Cell Culture DNA Mini Kit (QIAGEN, GER) and then treated with RNase A to produce the pure and RNA-free DNA. Meanwhile, and high-quality RNA was extracted from heart, stomach, liver, intestine, spleen, kidney, eye, brain, skin, ovary, and blood of *I. pollicaris* using the TRIzol Reagent Kit (Invitrogen, USA). The quality and concentration of DNA and RNA were evaluated by NanoDrop 1000 nucleic acid protein analyzer and NanoDrop 2000 ultramicro-spectrophotometer, respectively. Fragmentation buffer was applied to lyse the DNA and RNA into fragments with a suitable size. A high-quality Illumina library was constructed in accordance with the Illumina standard protocol (Illumina, USA), and a high-quality PacBio library was prepared using the PacBio library preparation kit (PacBio, USA) according to the manufacturer's protocol. Finally, the library was sequenced on the PacBio and Illumina HiSeq2500 platform. Additionally, A high-quality Hi-C library was constructed and then sequenced using the Illumina NovaSeq-6000 platform

The NECAT software [4] was utilized to pre-process, correct, trim, and *de novo* assemble of PacBio data. Hi-C reads containing adapter sequences or less than 50 bp in length were removed, and only PE Hi-C reads were retained. Bases with a quality score of less than 20 at both ends of the reads were eliminated. After aligning the Illumina and PacBio reads to the *I. pollicaris* genome sequence using HISAT2 [5], we employed BWA [6], minimap2 [7], BUSCO [8], samtools [9], picard [4] and GATK [10] software to evaluate the assembly effect of the genome. We obtained credible and nonredundant contigs interaction matrix using the HiCUP pipeline [11], and then immobilized contigs on chromosomes using the 3D-DNA pipeline [12]. Juicebox Assembly Tools [13] was applied to avoid the occurrences of chromosome inversion and translocation. Based on homologous prediction, *de novo* prediction, and EST prediction, we searched for the repetitive sequence of the *I. pollicaris* genome. Meanwhile, homolog homologous prediction, *de novo* prediction [14,15] and cDNA/EST prediction were combined to predict the location, structure, and function of *I. pollicaris* genes. Finally, four types of non-coding RNAs (including miRNA, tRNA, rRNA, and snRNA) were annotated by the tRNAscan-SE [16], Infernal [17], and BLASTN softwares.

To reveal the phylogenetics relationships between *I. pollicaris* and other species, we downloaded the protein-coding genes of *P. modestus, S. lalandi, O. latipes, U. bicinctus, C. lucidus, L. bergylta, E. moara, L. maculatus, S. aurata, M. mola, C. rostratus* from NCBI database (https://www.ncbi.nlm.nih.gov/). We first used OrthoMCL software [18] to obtained the single-copy orthologous genes common to all species. Subsequently, multiple alignment of single-copy orthologous was performed [19], and phylogenetic tree was ultimately constructed with RAxML software [19].

## Limitations

Not applicable.

## Ethics Statement

All experiments in the present study complied with the ARRIVE guidelines and were carried out in accordance with the U.K. Animals (Scientific Procedures) Act, 1986 and associated guidelines.

## CRediT Author Statement

**Tianxiang Gao and Fangrui Lou:** Conceptualization, Methodology, Software. **Tianxiang Gao and Fangrui Lou:** Data curation, Writing, Original draft preparation. **Yinquan Qu and Yiting wang:** Visualization, Investigation. **Tianxiang Gao:** Supervision. **Wenyu Li, Xingle Guo, Chenfeng Zhao:** Software, Validation. **Fangrui Lou:** Writing- Reviewing and Editing.

## Data Availability

The whole genome dataset of Ichthyscopus pollicaris (Original data)
